# Impact of a Machine Learning–Based Decision Support System for Urinary Tract Infections: Prospective Observational Study in 36 Primary Care Practices

**DOI:** 10.2196/27795

**Published:** 2022-05-04

**Authors:** Willem Ernst Herter, Janine Khuc, Giovanni Cinà, Bart J Knottnerus, Mattijs E Numans, Maryse A Wiewel, Tobias N Bonten, Daan P de Bruin, Thamar van Esch, Niels H Chavannes, Robert A Verheij

**Affiliations:** 1 Department of Public Health and Primary Care Leiden University Medical Center Leiden Netherlands; 2 Pacmed Amsterdam Netherlands; 3 Nivel Netherlands Institute for Health Services Research Utrecht Netherlands

**Keywords:** machine learning, ML, artificial intelligence, clinical decision support system, implementation study, information technology, urinary tract infections

## Abstract

**Background:**

There is increasing attention on machine learning (ML)-based clinical decision support systems (CDSS), but their added value and pitfalls are very rarely evaluated in clinical practice. We implemented a CDSS to aid general practitioners (GPs) in treating patients with urinary tract infections (UTIs), which are a significant health burden worldwide.

**Objective:**

This study aims to prospectively assess the impact of this CDSS on treatment success and change in antibiotic prescription behavior of the physician. In doing so, we hope to identify drivers and obstacles that positively impact the quality of health care practice with ML.

**Methods:**

The CDSS was developed by Pacmed, Nivel, and Leiden University Medical Center (LUMC). The CDSS presents the expected outcomes of treatments, using interpretable decision trees as ML classifiers. Treatment success was defined as a subsequent period of 28 days during which no new antibiotic treatment for UTI was needed. In this prospective observational study, 36 primary care practices used the software for 4 months. Furthermore, 29 control practices were identified using propensity score-matching. All analyses were performed using electronic health records from the Nivel Primary Care Database. Patients for whom the software was used were identified in the Nivel database by sequential matching using CDSS use data. We compared the proportion of successful treatments before and during the study within the treatment arm. The same analysis was performed for the control practices and the patient subgroup the software was definitely used for. All analyses, including that of physicians’ prescription behavior, were statistically tested using 2-sided *z* tests with an *α* level of .05.

**Results:**

In the treatment practices, 4998 observations were included before and 3422 observations (of 2423 unique patients) were included during the implementation period. In the control practices, 5044 observations were included before and 3360 observations were included during the implementation period. The proportion of successful treatments increased significantly from 75% to 80% in treatment practices (*z*=5.47, *P*<.001). No significant difference was detected in control practices (76% before and 76% during the pilot, *z*=0.02; *P*=.98). Of the 2423 patients, we identified 734 (30.29%) in the CDSS use database in the Nivel database. For these patients, the proportion of successful treatments during the study was 83%—a statistically significant difference, with 75% of successful treatments before the study in the treatment practices (*z*=4.95; *P*<.001).

**Conclusions:**

The introduction of the CDSS as an intervention in the 36 treatment practices was associated with a statistically significant improvement in treatment success. We excluded temporal effects and validated the results with the subgroup analysis in patients for whom we were certain that the software was used. This study shows important strengths and points of attention for the development and implementation of an ML-based CDSS in clinical practice.

**Trial Registration:**

ClinicalTrials.gov NCT04408976; https://clinicaltrials.gov/ct2/show/NCT04408976

## Introduction

### Background

The application of machine learning (ML) in health care is increasing. Previous studies have shown that using data from electronic health records (EHRs) can inform us about treatment effectiveness and outcomes in a real patient population, providing insight into unknown disease correlations in the process [1-3]. As current medical knowledge is often based on average results from studies in an isolated clinical setting, these data could fill important knowledge gaps in practice resulting from the fact that randomized controlled trials often use stringent selection criteria and therefore do not cover the complexity and variety of patients in everyday practice [4-9].

Most algorithms featured in academic research do not reach clinical practice nor are their performances evaluated prospectively [10-12]. This makes it challenging to assess the true added value of ML in health care as well as to formulate a scientific and societal vision on the balance between this added value and its pitfalls and risks. Finally, little research has been conducted on the interaction of a clinical decision support system (CDSS) with the end user, which greatly affects adoption and clinical results [13-16].

The treatment of urinary tract infections (UTIs) in primary care offers an opportunity to add clinical value to ML. UTIs are common and represent a significant health burden worldwide [17,18]. In the Netherlands, a UTI is the most frequent diagnosis in women consulting general practitioners (GPs), with an incidence rate of 125 per 1000 patient years and 19.6 per 1000 patient years for men in 2018 [19]. Uncomplicated UTIs often occur in young, healthy, and nonpregnant women. Certain host factors predispose to the development of a complicated course, including abnormalities of the urinary tract, male sex, diabetes mellitus, immune deficiency, or immune-compromising drugs [18,20,21]. The treatment guidelines for patients with UTIs were published by the Dutch College of General Practitioners (NHG). At the time of this research, guidelines published in 2013 were in place [22]. Most clinical trials on the treatment of UTIs that underpin the evidence in this guideline are conducted on female patients with uncomplicated infections; hence, the scientific evidence for clinically effective treatments with increased risk of complicated UTIs is limited [20-22]. GPs consider the lack of agreement as a problem for all key recommendations while using UTI guidelines [23].

The development of ML-based algorithms could facilitate better decision-making through the delivery of individualized recommendations based on real-world data on all types of patients, which could be beneficial in determining the optimal treatment for patients at risk for complicated UTIs [11].

### Supporting GPs With ML

Pacmed, a Dutch organization developing and implementing ML-based decision support in health care, developed, together with the consortium that conducted this research, a CDSS to aid GPs with the treatment choice for patients with a UTI. On the basis of the EHR data from UTI observations in the Nivel Primary Care Database, ML-based classifiers were constructed to estimate the probability of success of the 8 antibiotics commonly used for an individual patient with a UTI.

### Study Objective

In this study, we prospectively assessed the impact of the CDSS on the clinical results and prescription behavior of physicians. For this purpose, we compared the proportion of successful treatments before and during the implementation of the CDSS as well as the proportion of antibiotics chosen by the physician. By conducting an implementation study among GPs in 36 practices in the Netherlands, we aim not only to assess the impact of the software but also to study the interaction and adoption of the software. In doing so, we hope to identify general drivers and obstacles that positively impact health care with ML.

## Methods

### Study Design

This research was carried out following a routine practice-based prospective observational study design, in which 36 practices used the software (henceforth, the treatment practices) for a period of 4 months, starting in November 2017. A period of 4 months was chosen based on a power analysis of the primary outcome as well as the prevalence of patients with UTI in Dutch primary care. Treatment practices were mostly recruited at the care group level. This is a partnership between primary care practices to collaboratively organize care for chronic diseases. These groups also often decide to collectively participate in innovation projects such as this research, without consulting every individual GP or primary care practice. Physicians from all participating practices were trained on the responsible use of the software and were instructed to its intended use as supportive to their decisions (ClinicalTrials.gov NCT04408976).

### Ethics Approval

The study protocol was reviewed and determined to meet the requirements for exemption from the Ethics Committee (the Medical Ethical Committee) review under the Dutch Medical Research Involving Human Subjects Act (WMO) and to be in accordance with the Dutch Medical Treatment Act (WGBO) and the Dutch Data Protection Act (WBP, now AVG).

### The Clinical Decision Support Software: ML-Based Classifiers

The decision support system was developed through iterative consultation with multiple clinical stakeholders. A complete description of the model development and evaluation process is beyond the scope of this study. However, we provide some background information in the following paragraphs, highlighting the envisioned interaction with the end user in practice.

On the basis of the EHR data of patients who had at least one UTI between 2012 and 2014 and were >12 years, ML classifiers were constructed to estimate the probability of success for the 8 antibiotics commonly used for an individual patient with a UTI. In the potential absence of reliable UTI diagnosis data, we selected only patients who received antibiotic treatment for a UTI as reliably diagnosed patients in the data. Successful treatment was defined as a subsequent period of 28 days in which no new treatment was needed. The final data set for CDSS development contained 122,203 UTIs pertaining to 264 practices.

Owing to the anatomical differences between male and female patients with UTI, separate models were constructed for each sex. Fosfomycin was excluded as a treatment option in the clinical decision support system for male patients as this treatment is almost never used for male patients. The prediction would thus be of limited relevance, and the data set lacked sufficient data points to train a model. This approach resulted in 15 models in total: 8 for female patients and 7 for male patients.

The information presented by the ML classifiers was to be used in synergy with the existing experience and all other relevant sources of information. Hence, interpretability, clinical readability, and clinical relevance were prioritized in the development of the ML models. Decision trees were chosen as the classification method to allow for nonlinearities in the model while retaining interpretability. All 67 features that had been added as features to the classifiers were deemed medically important by the NHG guidelines issued at that time or had been indicated to affect treatment decisions, as discussed with medical experts [22]. These variables include patient characteristics, such as the presence of diabetes, pregnancy, indications of tissue invasion, dysfunctional urinary tracts, medical UTI history, and the treatment associated with these episodes. Other predictive features that were more difficult to interpret medically were also excluded. The classifiers were constructed using a scikit-learn pipeline, including missing value imputation, feature scaling, and L1 feature selection [24]. Hyperparameters were optimized using 10-fold cross-validation, and the model performance was evaluated using a cross-validated area under the receiver operator curve. This approach yielded modest model performance in terms of area under the curve (averaging around 0.6 over all models).

Although the classifiers were not able to predict with high accuracy which treatments would certainly (not) be successful, the models allowed for distinguishing patients with a relatively high risk of unsuccessful outcomes from patients with a low risk of unsuccessful treatment. More importantly, for a single patient, the models distinguished between treatments with a relatively high risk of unsuccessful outcomes and treatments with a medium or low risk of unsuccessful outcomes. Thus, although a substantial part of the outcome variation is unexplained, we expected the use of the model’s predictions for treatment decisions to positively affect treatment outcomes.

The medical soundness and relevance of the decision trees were confirmed by multiple clinical experts inspecting the features and resulting models through a long list of clinical hypotheses on the practical performance of treatments for different patient groups. Moreover, before the implementation study, a (the Medical Ethical Committee’) passive model validation was performed. Showing the predictions as well as the support information of the models for patients with UTI treated less than a month ago by their physician, we validated the usability, relevance, reliability, and interpretability of the information presented. These rounds of validations with medical experts convinced us that the models were reliable and could add value to clinical practice.

### The Clinical Decision Support Software: User Interface

**Figure 1:**
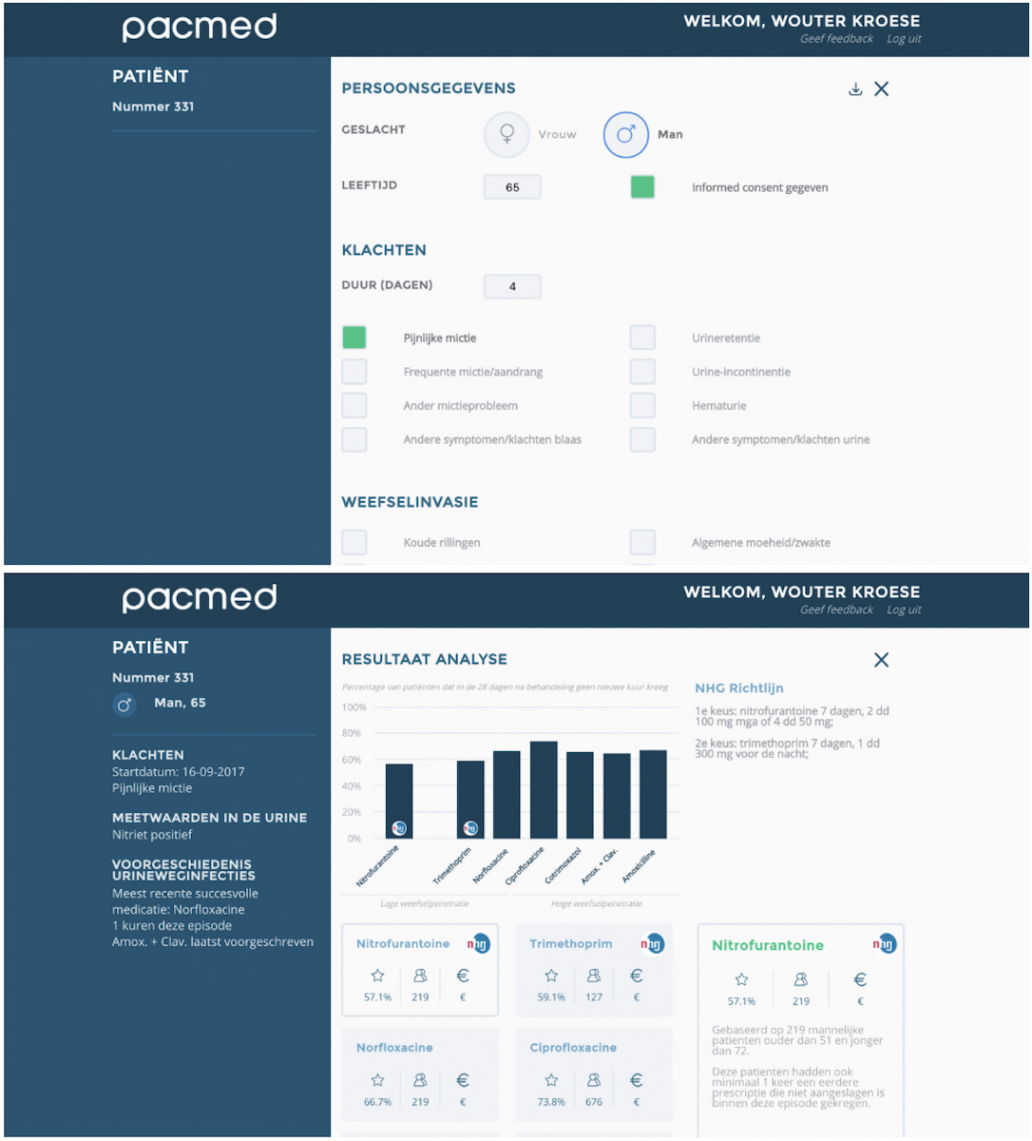
Decision support software: interface to enter patient characteristics (top); presentation of expected outcomes and NHG (Dutch College of General Practitioners) guidelines (bottom).

The software interface was developed in close collaboration with several primary care physicians through user tests and expert groups. The CDSS was not integrated into the EHR of GPs, so users were requested to enter patient characteristics into the web-based software. To invite the end user to interpret the information thoroughly and in an unbiased manner, antibiotics were always presented in the same order, independent of the probability of treatment success. Users were provided with a bar chart showing the estimated outcomes for the relevant treatment options based on similar patients within the database (Figure 1).

The algorithms in the CDSS presented the expected outcomes as well as the necessary support information for the physician at the time of the treatment decision. Owing to the choice of decision tree classifiers, we were able to follow the characteristics of an individual patient through the decision tree nodes and share with the physician the characteristics of the sample which was used to predict the outcome of a treatment. This information, such as the age range of these patients and other clinically relevant features, can be retrieved by clicking on the relevant treatment.

We chose not to display the models themselves because the large number of features and the different models would have been confusing. We did not add CIs around the predictions in the user interface. Calculating and presenting a CI around a probabilistic prediction is not straightforward, and this complexity could have been confusing or incorrectly interpreted.

In addition to the presentation of the expected outcomes, the relevant part of the 2013 NHG guidelines was also presented. All 8 antibiotics from the NHG recommendations for patients with a UTI are shown, although for female patients without signs of tissue invasion, after expert consultation, it was decided not to show antibiotics with high tissue penetration as treatment options, as other treatments should be considered and tried first in most instances. Finally, GPs were instructed to use the software only for patients >12 years and to assess the information presented together with all other information they deemed relevant in treating patients with a UTI.

### Selection of Control Practices

Control practices were identified from a pool of 129 potential control practices in the Nivel database through a propensity score-matched augmented control procedure. As shown in previous research, these matching methods can be used to construct an artificial control group for trials by matching treatment and control units that are similar in terms of their observable characteristics [25,26]. As practice characteristics are most informative in the way patients are being treated, propensity score-matching was performed at the aggregated practice level. The total number of patients per practice and their average age were the characteristics used to construct the propensity. Matching was performed using a caliper of 0.05.

The treatment practices were matched with 29 control practices. Data from all patients with a UTI in these practices were analyzed. This resulted in 4998 observations of patients with a UTI in the treatment practices before the pilot started and 3422 observations during the pilot. The control practices resulted in 5044 observations before and 3360 observations during the pilot.

### Preparation of Study Data

#### Nivel Primary Care Database

All analyses were performed using the Nivel Primary Care Database, containing structured EHR data from 530 GP practices in the Netherlands. A selection was made so that the data set only contained the data of all patients with at least one UTI during this study.

The data request was approved by all necessary and appropriate bodies from the Nivel Governance-Document Nivel Primary Care Database [27] under number NZR00317.030. The use of the data for this specific research was in accordance with all relevant Dutch and European laws and legislations.

The study included all patients who had at least one indication of UTI symptoms, indicated by the International Classification of Primary Care codes U70 (acute pyelonephritis), U71 (cystitis), U72 (nonspecific urethritis), U01 (painful micturition), or U02 (frequent micturition) and were prescribed antibiotic treatment for this UTI.

We used data from 2 periods. The first period consisted of the time before the implementation study (week 16 until week 4), and the second period consisted of the time during the implementation period (weeks 0 to 20). Within these 20 weeks, the software was used for different periods of 16 weeks. Owing to the defined outcome measure of treatment success that requires patients not to receive another treatment for UTI within 28 days, an additional 4 weeks were added to ensure that the treatment outcome of these patients was also captured within the analysis.

The prescribed treatment for UTI was directly recorded in the EHR system of the GP. Background information about the patient and their comorbidities consisted of a combination of diagnoses and symptom codes and the prescription of other medications related to these comorbidities.

#### Pacmed Use Database

Through use of physicians and their assistants, data on patient characteristics and chosen treatments were generated using Pacmed software. These data were generated with informed consent from the treated patients.

The patients present in this database were those for whom we were certain that the software had been used. Therefore, we attempted to identify these *Pacmed patients* in the Nivel database. However, because all identifiable personal data were removed for both the Pacmed software and the Nivel database, it was not possible to match these 2 databases directly.

Instead, identification took place iteratively using a sequential matching procedure. A single matching approach failed because of practical challenges resulting from the nature of the data sets. The Nivel database was generated automatically from all the different information systems used in the participating practices, resulting in a data set with subtle differences between the practices. The Pacmed data consist of data directly resulting from the use of the CDSS. Attempting to match the databases through a single matching procedure based on practice location, gender, date of birth, and the data of consultation failed, as we assessed it as very likely that the Pacmed software had been used days after the first visit or at a second visit. Therefore, another approach was used where patient identification was performed iteratively through a sequential matching procedure, in such a way that after an initial merge, unique matches that were found were removed and the matching with a new variable constellation for the remaining patients was continued.

First, an effort was made to identify patients from Pacmed in the raw EHR data provided by Nivel. Thereafter, additional matching was performed for patients in the processed Nivel data set. Variables that were matched based on the raw Nivel data included age, sex, date of birth, and date of consultation. In addition, age categories, day intervals around the date of consultation, prescribed medication, and comorbidities were constructed. All matches included gender and practice postal codes.

### Primary and Secondary Outcomes

The primary outcome of interest was the difference in the proportion of successful treatments between the periods before and during the study. Successful treatment was defined as a period of 28 days after the initial treatment, during which no new treatment was needed. This outcome definition was constructed using GP expert groups and a meticulous analysis of the impact of different definition choices.

The analyses of the primary outcomes were repeated for subgroups based on sex and age, if the patients had diabetes and if UTI was complicated. In addition, to directly compare the differences between the treatment and control arms, the primary outcome was compared between the groups during the implementation study.

A total of 2 sensitivity analyses were performed to test the robustness of primary outcomes. First, to exclude potential temporal effects, the same test for the primary outcome of interest was performed for control practices. Second, the proportion of successful treatments for patients for whom the software had certainly been used was compared with the proportion of successful treatments for patients in the treatment practices before the implementation study.

Finally, the prescription behavior of the physicians was analyzed to determine whether there was a statistically significant difference in prescribed antibiotics between the treatment and control practices before and during the implementation study period. This analysis was repeated for observations for which we were sure that the software had been used. Specifically, we were interested in the difference in the proportion of high tissue penetration antibiotics chosen by physicians when presented with the expected outcomes of treatments.

### Statistical Analysis

#### Power Analysis

We conducted a power analysis to gauge the required number of observations of patients with UTIs to detect small effect sizes (Cohen effect size of 0.1) of the intervention [28]. Patients were clustered within a practice, making them more likely to be treated or respond similarly within that practice. To account for this clustering, sample sizes were adjusted using an inflation factor [25]. The inflation factor is a function of the intracluster correlation coefficient and average cluster sizes per practice. However, the mean average cluster size (the number of patients with a UTI) per practice was 72.7, and practices showed large variations in average cluster sizes (SD 98.3, range 10-325). Therefore, along with the inflation factor, a cluster variation coefficient was calculated for the outcome variables. Thus, the reported sample size was adjusted, including the intracluster correlation and cluster variation coefficients. The desired sample size was calculated to be at least 851 at a power of 0.8 and a type 1 error rate of 0.05 to detect small effects.

#### Outcome Statistics

A total of 2 sample *z* tests with an *α* level of .05 were used to test the statistical significance of the primary outcome analysis, both the sensitivity analyses and the prescription behavior analyses. To test the significance of the subgroup analyses for the primary outcome measure, additional *z* tests were used. To determine whether the differences were statistically significant and to avoid the inflation of type 1 errors, Bonferroni corrections were applied. Differences with a *P* value <.006 (0.05/9) were determined to be statistically significant. To further compare the primary outcomes across different subgroups, the relative risk ratio was used [29].

## Results

### Patient Population

The Pacmed use database contained 1689 unique patients, of which 734 (43.46%) unique individual patients were identified in the Nivel database through the sequential identification procedure. This is the number of patients for whom we can be certain that the software was used.

**Figure 2:**
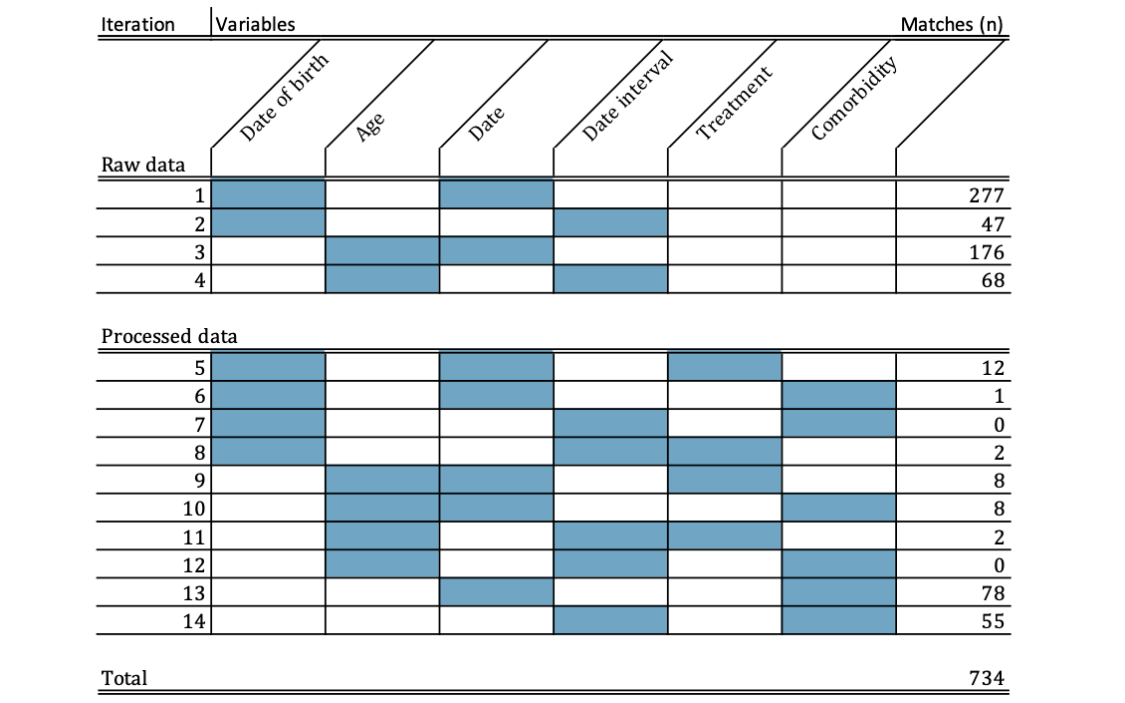
The number of matches found through the sequential matching procedure.

Figure 2 shows the variables used in the sequential matching procedure and the number of matches found in each iteration. Table 1 displays the patient characteristics for the cohort in treatment and control practices during the implementation study and specifies the characteristics of the patients identified from the Pacmed use database.

**Table 1:** Patient characteristics of the observations in treatment and control practices during the implementation study.

Characteristic			Proportion of patients in treatment practices observations (n=3422)		Proportion of patients identified in Pacmed Clinical Decision Support System observations (n=1121)		Proportion of patients in control practices observations (n=3360)
Sex (female)			0.88		0.92		0.86
**Age (years)**							
	<30	0.17		0.15		0.17	
	30-50	0.21		0.18		0.20	
	50-70	0.36		0.39		0.29	
	>70	0.26		0.28		0.33	
Diabetes			0.08		0.09		0.13
UTI^a^ with tissue invasion^b^			0.11		0.13		0.12
Complicated UTI^c^			0.12		0.13		0.13

^a^UTI: urinary tract infection.

^b^A UTI with tissue invasion was defined as a UTI with which (a combination of) the International Classification of Primary Care codes associated with tissue invasion-related symptoms were registered (A02, A03, A04, and A05).

^c^Complicated UTI is defined as a UTI with tissue invasion or a simultaneous pyelonephritis or prostatitis episode, International Classification of Primary Care codes U70 and Y93, respectively.

### Evaluation Outcomes

#### Primary Outcome and Sensitivity Analyses

The proportion of successful treatments increased significantly from 75% to 80% in treatment practices (*z*=5.47; *P*<.001). In the control practices, no significant change in outcomes was observed during the same period (76% before and 76% during the pilot, *z*=0.02; *P*=.98). The proportion of successful treatments during the study was 83% for the observations of which we are certain that the software had been used. This was a statistically significant difference, with 75% of successful treatments before the study in the treatment practices (*z*=4.95; *P*<.001). The comparison of the primary outcome between the control practices and the treatment practices during the implementation study also showed a significant difference (76% for the treatment practices and 80% for the control practices, *z*=4.86; *P*<.001).

The change in outcome has been specified for subgroups based on sex, age, comorbidities (diabetes) and whether the UTI was complicated in Table 2. In this analysis, the increase in outcomes was statistically significant for female patients and patients >70 years.

**Table 2:** Test statistics of primary outcome or several patient subgroups in the treatment practices observations before (n=4998) and during (n=3422) the study.

Subgroup		Proportion of successful treatments before study	Proportion of successful treatments during study	Risk ratio	*P* value
**Sex**					
	Female (n=3008)	0.76	0.81	1.07	<.001
	Male (n=414)	0.72	0.78	1.08	.01
**Age (years)**					
	<30 (n=580)	0.81	0.86	1.06	.01
	30-50 (n=719)	0.80	0.83	1.04	.05
	50-70 (n=1227)	0.76	0.79	1.04	.05
	>70 (n=896)	0.70	0.76	1.09	<.001^a^
Complicated UTI^b,c^ (n=404)		0.72	0.79	1.10	.03
Diabetes (n=275)		0.71	0.79	1.11	.03

^a^Significant Bonferroni adjusted *P* values (.05/9).

^b^UTI: urinary tract infection.

^c^A complicated UTI is defined as a UTI with tissue invasion or a simultaneous pyelonephritis or prostatitis episode, International Classification of Primary Codes U70 and Y93, respectively.

#### GP Prescription Behavior

In the treatment practices as well as in the control practices, there was no significant difference in the proportion of high tissue penetration antibiotics prescribed between the period prior and during the implementation study. Table 3 has additional information on the choice of treatment before and during the study period. As it is known that there are sex differences in prescribed medications owing to differences in underlying etiology, the same table is shown for both sexes. Table 4 shows the same information for the observations for which we were sure that the software was used.

**Table 3:** Proportion of medication prescribed before and during implementation study.

				Treatment practices							Control practices						
				Before	During	Delta %		*P* value		Before			During		Delta %		*P* value
**All patients**				n=4998	n=3422					n=5044			n=3360				
	**Antibiotics with low tissue penetration**		0.79		0.80	0.01	.37		0.79			0.77		−0.02		.02	
		Nitrofurantoin	0.59		0.59	−0.01	.56		0.60			0.57		−0.02		.03	
		Fosfomycin	0.13		0.13	0.00	.70		0.14			0.15		0.01		.24	
		Trimethoprim	0.06		0.06	0.00	.58		0.05			0.04		−0.01		.11	
		Norfloxacin	0.01		0.02	0.02	.001		0.00			0.00		0.00		.63	
	**Antibiotics with high tissue penetration**		0.21		0.20	−0.01	.37		0.21			0.23		0.02		.02	
		Ciprofloxacin	0.16		0.14	−0.02	.04		0.13			0.15		0.01		.09	
		Augmentin	0.03		0.03	0.00	.44		0.04			0.04		0.00		.59	
		Sulfamethoxazole and trimethoprim	0.02		0.02	0.00	.65		0.02			0.03		0.01		.005	
		Amoxicillin	0.01		0.01	0.00	.45		0.01			0.01		0.00		.58	
**Female patients**				n=4361	n=3008					n=4439			n=2881				
	**Antibiotics with low tissue penetration**		0.84		0.84	0.00	.99		0.84			0.83		−0.01		.12	
		Nitrofurantoin	0.62		0.61	−0.01	.39		0.63			0.62		−0.02		.11	
		Fosfomycin	0.14		0.14	0.00	.75		0.15			0.16		0.01		.12	
		Trimethoprim	0.06		0.06	0.00	.99		0.05			0.04		−0.01		.09	
		Norfloxacin	0.01		0.01	0.01	.006		0.00			0.00		0.00		.69	
	**Antibiotics with high tissue penetration**		0.16		0.16	0.00	.99		0.16			0.17		0.01		.12	
		Ciprofloxacin	0.12		0.11	0.00	.66		0.10			0.10		0.00		.61	
		Augmentin	0.02		0.02	0.00	.55		0.10			0.10		0.00		.61	
		Sulfamethoxazole and trimethoprim	0.01		0.01	0.00	.98		0.02			0.02		0.01		.01	
		Amoxicillin	0.01		0.01	0.00	.77		0.01			0.01		0.00		.39	
**Male patients**				n=637	n=414					n=605			n=478				
	**Antibiotics with low tissue penetration**		0.44		0.49	0.05	.14		0.44			0.43		−0.01		.77	
		Nitrofurantoin	0.36		0.37	0.01	.86		0.33			0.33		−0.01		.84	
		Fosfomycin	0.03		0.03	0.00	.94		0.06			0.06		−0.00		.92	
		Trimethoprim	0.03		0.05	0.02	.08		0.04			0.04		0.00		.97	
		Norfloxacin	0.02		0.04	0.02	.09		0.01			0.01		0.00		.70	
	**Antibiotics with high tissue penetration**		0.56		0.51	−0.05	.14		0.56			0.57		0.01		.77	
		Ciprofloxacin	0.44		0.35	−0.09	.003		0.39			0.42		0.02		.41	
		Augmentin	0.07		0.08	0.01	.52		0.09			0.06		−0.02		.13	
		Sulfamethoxazole and trimethoprim	0.03		0.05	0.01	.37		0.06			0.07		0.01		.36	
		Amoxicillin	0.01		0.02	0.02	.05		0.02			0.01		−0.01		.50	
**Patients with complicated UTI^a,b^**				n=365	n=404					n=369			n=433				
	**Antibiotics with low tissue penetration**		0.58		0.62	0.05	.19		0.62			0.61		−0.01		.74	
		Nitrofurantoin	0.42		0.43	0.01	.80		0.43			0.36		−0.06		.07	
		Fosfomycin	0.10		0.10	0.00	.91		0.10			0.21		0.12		.92	
		Trimethoprim	0.05		0.06	0.02	.37		0.06			0.03		−0.04		.02	
		Norfloxacin	0.01		0.03	0.02	.01		0.03			0.00		−0.03		.003	
	**Antibiotics with high tissue penetration**		0.42		0.38	−0.05	.19		0.38			0.39		0.01		.74	
		Ciprofloxacin	0.30		0.22	−0.08	.01		0.22			0.22		0.00		.97	
		Augmentin	0.07		0.08	0.01	.77		0.08			0.10		0.02		.26	
		Sulfamethoxazole and trimethoprim	0.03		0.05	0.02	.11		0.05			0.05		0.00		.93	
		Amoxicillin	0.02		0.02	0.00	.76		0.02			0.02		−0.01		.53	
**Patients with diabetes**				n=355	n=275					n=359			n=429				
	**Antibiotics with low tissue penetration**		0.70		0.72	0.01	.74		0.75			0.68		−0.07		.02	
		Nitrofurantoin	0.47		0.41	−0.06	.14		0.52			0.43		−0.09		.01	
		Fosfomycin	0.14		0.14	0.00	.92		0.17			0.18		0.01		.65	
		Trimethoprim	0.06		0.10	0.04	.10		0.06			0.06		0.01		.67	
		Norfloxacin	0.03		0.06	0.04	.03		0.01			0.01		0.00		.83	
	**Antibiotics with high tissue penetration**		0.30		0.28	−0.01	.74		0.25			0.32		0.07		.02	
		Ciprofloxacin	0.25		0.22	−0.03	.43		0.17			0.21		0.04		.16	
		Augmentin	0.03		0.04	0.00	.86		0.04			0.04		0.00		.88	
		Sulfamethoxazole and trimethoprim	0.01		0.02	0.00	.69		0.02			0.05		0.03		.03	
		Amoxicillin	0.00		0.01	0.01	.08		0.01			0.01		0.01		.45	
**Patients with age >70 years**				n=1599	n=896					n=1721			n=1122				
	**Antibiotics with low tissue penetration**		0.70		0.72	0.02	.31		0.74			0.70		−0.04		.02	
		Nitrofurantoin	0.45		0.43	−0.03	.20		0.48			0.45		−0.03		.11	
		Fosfomycin	0.16		0.17	0.01	.48		0.20			0.19		−0.01		.74	
		Trimethoprim	0.07		0.07	0.01	.47		0.06			0.06		−0.01		.47	
		Norfloxacin	0.02		0.05	0.03	.001		0.00			0.01		0.00		.58	
	**Antibiotics with high tissue penetration**		0.30		0.28	−0.02	.31		0.26			0.30		0.04		.02	
		Ciprofloxacin	0.24		0.21	−0.03	.06		0.17			0.20		0.03		.03	
		Augmentin	0.03		0.04	0.01	.39		0.05			0.03		−0.02		.03	
		Sulfamethoxazole and trimethoprim	0.02		0.03	0.01	.32		0.02			0.04		0.02		.004	
		Amoxicillin	0.01		0.01	0.00	.75		0.01			0.02		0.01		.27	

^a^UTI: urinary tract infection.

^b^A complicated UTI is defined as a UTI with tissue invasion or a simultaneous pyelonephritis or prostatitis episode, International Classification of Primary Care codes U70 and Y93, respectively.

**Table 4:** Proportion of medication prescribed before and during implementation study for all patients, for the observations identified from the Pacmed use database.

			Treatment practices			
			Before (n=4998)	During (identified; n=1121)	Delta %	*P* value
**Antibiotics with low tissue penetration**			0.79	0.81	0.02	.15
	Nitrofurantoin	0.59		0.53	−0.07	<.001
	Fosfomycin	0.13		0.16	0.03	<.001
	Trimethoprim	0.06		0.08	0.02	.01
	Norfloxacin	0.01		0.04	0.03	<.001
**Antibiotics with high tissue penetration**			0.21	0.19	−0.02	.15
	Ciprofloxacin	0.16		0.13	−0.03	.01
	Augmentin	0.03		0.03	0.00	.53
	Sulfamethoxazole and trimethoprim	0.02		0.02	0.00	.39
	Amoxicillin	0.01		0.01	0.01	.02

## Discussion

### Principal Findings

The most important result of this study is that the introduction of the CDSS as an intervention in the 34 treatment practices was associated with improved treatment success for patients with UTI. The percentage of successful treatments in the patient population increased from 75% before implementation of the CDSS to 80% during the implementation period. Next to a significant increase in treatment outcome within the treatment arm, the difference between treatment and control group was also significant (76% in control practices and 80% in treatment practices during the study). These control practices were selected through propensity score-matching and were similar at baseline. This resulted in control practices that, at the time of the study, had comparable patient populations with UTI.

To assess whether the increase in treatment success was due to the CDSS presented in this study, we performed 2 sensitivity analyses. First, the software was not used for all patients in the Nivel database. Therefore, we sought to identify patients from the Pacmed use database in the Nivel database. We had been able to identify more than half of the patients in the Pacmed use database in the Nivel database. The association of the increase in treatment success with the introduction of the intervention was strengthened by the fact that the increase in clinical outcome was even higher and statistically significant (83%) for the patients for whom we were sure that the software had been used. The significance of this test must be seen from the perspective that the populations are not identical, and that there is a potential selection bias in the patient population in the CDSS database. However, Table 1 shows comparable prevalence among the relevant clinical subgroups.

Second, we assessed whether the increase in treatment success was due to temporal effects, namely, the spontaneous improvement of treatment success for all practices, independent of the introduction of the CDSS. In the control practices, no significant increase in the proportion of successful treatments was observed.

Finally, the increase in treatment success did not seem to have been caused by an increase in the prescription of high tissue penetration antibiotics. On an average, for female and male patients, we did not observe a significant difference in the proportion of antibiotics with high tissue penetration. We observed a significant increase in the prescription of norfloxacin in female patients.

### Behavior Change Within the Treatment Practices

An increase in treatment success was observed for multiple subgroups. In this analysis, only the results for female patients and patients aged >70 years were found to be statistically significant. Other subgroups with a noteworthy increase in outcomes included male patients, patients with complicated UTIs, and patients with diabetes. The reason that the increase in outcome cannot be deemed significant in this analysis is presumably partly owing to the sample sizes of these subgroups, as opposed to lower effect sizes. In particular, most clinical trials on the treatment of UTIs, supporting the evidence in the Dutch GP guidelines at the time, were conducted in female patients with uncomplicated infections [20-22]. One could then expect that an ML-based CDSS, aiming to fill knowledge gaps by learning from more complex patients in practice, would be most valuable for patient groups that are now understudied.

Within these subgroups, although not statistically significant, we observed an indication of behavioral change in the treatment arm. For all these subgroups (male, patients with diabetes, patients with a complicated UTI, and patients >70 years), the proportion of norfloxacin treatments doubled, which was not observed in the control practices. Norfloxacin was not recommended as a treatment option for all subgroups in the NHG guideline.

The NHG guidelines at the time recommended nitrofurantoin as the first choice for male and diabetic patients, with trimethoprim as the second choice. Trimethoprim prescriptions almost doubled for both subgroups only in the treatment practices. For patients with a complicated UTI, we observed a decrease in ciprofloxacin treatment only in the treatment practices, although ciprofloxacin was the first recommended treatment in the NHG guidelines at the time. Using Bonferroni adjusted *P* values, the difference in prescription behavior was not deemed to be statistically significant, and the effect of this indicated behavior change on clinical outcome should serve as a hypothesis for future research. However, it should be noted that it is unlikely that the increase in outcomes is (solely) owing to better guideline adherence. The analysis of behavioral changes for the CDSS patients identified in the Nivel database confirms this insight, with a significant decrease in nitrofurantoin treatments, which is the first recommended treatment option for almost all patient groups in the guideline, and a significant increase in norfloxacin treatments.

However, many other unmeasured factors could have improved patient outcomes independent of the information presented by the CDSS. Among other things, knowing to participate in the trial could have led to a better diagnosis and more conscious treatment choice, independent of the relevance or value of the CDSS.

### Strengths

Only by integrating the knowledge in the clinician’s workflow and evaluating the impact prospectively can we truly assess the potential added value of a new technology such as ML in today’s health care system. A strength of this study is the fact that we developed a CDSS that, through its accessibility, was often used by the participating physicians to enable us to analyze the difference in our chosen outcome on a scale large enough for the results to be statistically significant. A more in-depth study on the use and perceived accessibility of physicians is in preparation.

In the design of the software, the transparency of the underlying technology was key to ensuring its usability. The algorithms that form the intelligence of the software were deliberately chosen to be interpretable and understandable models for the end user as well as for the physicians who were part of the development. In addition, the resulting predictions of treatment success presented in the CDSS during the study were accompanied by supporting information regarding the patient characteristics on which the predictions were based. Finally, by presenting the relevant subsection of the active NHG guidelines, we presented the necessary context information to assess all sources of information equally, enabling the physician to combine these sources together with their experience, expertise, and intuition to make the best decision for the patient.

The execution of sensitivity analyses to assess the robustness of the association between treatment success and the intervention of the CDSS is another strength of this study. We had access to EHR from the Nivel database of treatment practices as well as from control practices, enabling us to exclude temporal effects. The fact that we had access to the Pacmed use database made it possible to specifically analyze the subgroup of patients for whom we were certain that the tool had been used.

We observed that for patients in whom the tool had been used, the outcome improvement seemed more pronounced compared with the rest of the patients in the treatment practices. This suggests a clear added value of the CDSS tool itself, rather than the mere fact that more attention was paid to UTI in these practices. Next, it is noteworthy that we were able to assess the adoption rate of the software and relate it to the outcome of interest. Only by doing so is it possible to evaluate the expected impact in relation to the (financial) investment needed to develop, implement, integrate, monitor, and continuously improve complex technologies such as ML for physicians [14,15].

### Limitations

To assess the impact of the CDSS on treatment success, we chose several measurements to ensure the robustness and reliability of our outcome measure. To ensure that the patients included were indeed affected by UTI, we selected only patients on having received antibiotic treatment rather than using only the diagnosis code as the selection criterion. However, a more specific diagnosis of a UTI can be made through laboratory data, which have not been used, as laboratory test results were not recorded in the EHR data for many UTI patients. In addition, there was a selection bias in selecting only patients who received antibiotic treatment. However, this selection bias was the same for the treatment and control practices for this study.

Furthermore, treatment success is indirectly derived from the information systems of GPs and is used as a proxy for clinical examination. This method is similar to the algorithms used to construct disease episodes based on EHR data [30]. A similar methodology was followed, defining treatment success as a subsequent period of 28 days, where no new treatment was needed, indicating a reduced risk for treatment failure or relapse. Possible flaws concerning therapy compliance cannot be mitigated using these data. Information about the resolution of complaints or bacterial clearance, and thus, decisive knowledge on treatment success, was absent. In addition, it is likely that unnecessary antibiotic prescriptions were considered successful based on our definition of treatment success. However, the potential flaw in our outcome definition was consistent with treatment and control practices. Moreover, we have no indication that the use of the CDSS resulted in more unnecessary prescriptions (and with that positively influenced the primary outcome of this research). In both the treatment and control practices, the total number of antibiotic prescriptions decreased by almost identical proportions (from 4998 to 3422 in the treatment practices [–32%] and from 5044 to 3360 in the control practices [–33%]).

In addition, negative effects, side effects, or impacts on general resistance to treatments were not investigated in this study. These are, of course, factors that should impact the evaluation of a CDSS of this kind in practice. However, we excluded antibiotics with high tissue penetration in the software as treatment options, as we were aware of the potential negative effects of these treatments. This might have contributed to the fact that we did not observe a significant increase in these proportions of treatments prescribed during the study. Furthermore, it can be expected that the side effects of the treatments were considered by GPs in the treatment and control arms of the study. GPs were actively advised to choose a treatment based on all the information they deemed relevant, not only the information presented through the CDSS. Therefore, as we expect GPs to include knowledge on the side effects and negative effects of treatments in their decision-making, we mitigate the impact of this information being absent in the CDSS.

Finally, we were unable to relate the significant increase in patient outcomes to significant behavior change by physicians in treatment practices. The analyses of behavior change could potentially have been done more thoroughly if we had been able to match more patients between the Pacmed data and Nivel database. Out of 1200, only 734 (61.16%) patients could be matched owing to an underestimated complexity in matching these databases. In hindsight, a pseudonymized patient identifier would have enabled us to match a significantly higher number of patients in the Nivel database, if not all, from the CDSS data. The most important underestimation of this complexity was the incorrect assumption that participating physicians and assistants would always use the software during the first consultation with the patients. As this was not the case, it was difficult to identify patients based on their characteristics as well as the time they had entered into the software. We strongly recommend extensive research on the care paths of patients in multiple clinical institutions to match the implementation study design with these care paths. Another effort to further understand the behavior change and thus the impact of the CDSS would be to have expert groups and extensive surveys on how the software impacted the decision-making of GPs and assistants at an individual level.

### Relevance and Future Directions

This research brings important knowledge to the research field of responsibly implemented ML-based decision support systems with clinical relevance. In particular, most published algorithms do not reach the frontline of clinical practice nor are they validated prospectively [10,11]. To make this technology live up to its great promises, prospective validation is needed, resulting in high-quality protocols for the responsible development and deployment of ML in health care [11,14,31-34].

Unfortunately, there is very little scientific discussion on the responsible evaluation of the CDSS to assess its impact and validity once it has been implemented. Nevertheless, only by evaluating its impact on relevant outcomes, while integrated in the clinical workflow, the potential and risks can be fully understood [15,35]. One of the greatest barriers to achieving impact in practice is the adoption of the CDSS by the clinician as an end user [15,33]. Nevertheless, studies often fail to assess (or report on) the impact of the technology on the users and their workflow [13,14,16]. Therefore, it is even more important to have protocols in place to thoroughly analyze the behavior change, assess its robustness, and compare the outcomes for groups for which the tool has certainly been used with those that one is uncertain about.

### Conclusions

The introduction of the CDSS as an intervention in the 36 treatment practices was associated with a statistically significant improvement in treatment success for patients with a UTI, namely, an increase from 75% to 80% successful treatments. The 2 sensitivity analyses enabled us to present this result with greater robustness. First, temporal effects were excluded by evaluating treatment success in the same period for a group of control practices selected through a propensity score-matching procedure. Second, analyzing the subgroup for whom we were certain the software had been used strengthened the association of an increase in successful treatment with the presentation of the CDSS in the treatment arm.

This study shows some important strengths and points of attention in the design and development of clinical decision support software as well as a thorough evaluation of its clinical impact in practice. Further research is needed on the interaction of ML-based clinical decision support software with end users to assess the potential impact of this technology for patients and physicians, and to develop concrete and objective guidelines to perform this research responsibly.

## Abbreviations

CDSS: clinical decision support system
EHR: electronic health record
GP: general practitioner
LUMC: Leiden University Medical Center
ML: machine learning
NHG: Dutch College of General Practitioners
UTI: urinary tract infection
